# First Detection of a Novel Reassortant Avian Influenza A(H5N6) Clade 2.3.2.1c Virus, Isolated from a Wild Bird in China

**DOI:** 10.1128/MRA.00797-19

**Published:** 2019-09-05

**Authors:** Tao Zhang, Ruiyun Li, Gaodong Zhu, Jianyu Chang, Bing Xu

**Affiliations:** a Ministry of Education Key Laboratory for Earth System Modeling, Department of Earth System Science, Tsinghua University, Beijing, China; b MRC Centre for Global Infectious Disease Analysis, Department of Infectious Disease Epidemiology, School of Public Health, Faculty of Medicine, Imperial College London, London, United Kingdom; c Suichuan Forestry Bureau, Jiangxi, China; d College of Veterinary Medicine, China Agricultural University, Beijing, China; DOE Joint Genome Institute

## Abstract

We report the first isolation of a reassortant clade 2.3.2.1c avian influenza A(H5N6) virus isolated from a wild bird sample in Jiangxi, China, in 2016. Sequence analyses indicated that this virus most likely evolved from Eurasia-derived H5N1 and H6N6 viruses through frequent interactions at the wild-domestic bird interface.

## ANNOUNCEMENT

Avian influenza virus (AIV) is a single-stranded segmented negative-sense RNA virus classified in the Alphainfluenzavirus genus in the family *Orthomyxoviridae* (1). The hemagglutinin (HA) gene of highly pathogenic avian influenza (HPAI) A(H5) viruses has undergone continuous evolution, generating emerging reassorted subtypes and clades (2). Among these variants, two relatively new clades, i.e., 2.3.4.4 and 2.3.2.1c, predominately shaped the current viral evolution and transcontinental expansions (3, 4). It is remarkable that clade 2.3.4.4 involves multiple subtypes, including H5N1, H5N2, H5N6, and H5N8 (2). In contrast, all clade 2.3.2.1c viruses, including the novel Sanmenxia clade 2.3.2.1c-like H5N1 virus isolated in a wild bird, belong to H5N1 (5).

Here, we provide a report on the first detection and nearly complete genome sequence of a novel reassortant clade 2.3.2.1c H5N6 sample isolated from a wild bird in China. A total of 488 tracheal and cloacal swab samples were collected during a routine bird survey in Suichuan County, Jiangxi Province, which were preserved in a sample solution in the fridge (4°C) and subsequently shipped to a laboratory and stored frozen at −80°C. From these samples, 89 and 85 samples were taken from Streptopelia decaocto and yellow-legged button quail, respectively. Virus isolation using these specimens was conducted in 9- to 11-day-old specific-pathogen-free embryonated chicken eggs. The viral RNAs were extracted from allantoic fluid of 13 samples with hemagglutination activity using an RNeasy minikit (Qiagen, Hilden, Germany). The SuperScript III reverse transcriptase (RT) PCR kit (Invitrogen, USA) was used for the reverse transcription.

The subtype of each of the 13 positive samples was first determined using PCR of a marker gene (6, 7). Seven out of 13 samples were identified as H5N6 strains. All segments of the H5N6 strains were amplified by using a Phusion high-fidelity PCR system (New England BioLabs, Ipswich, MA, USA), adhering to the manufacturer’s guide (8). Sequencing of each segment was subsequently performed as individual amplicons using the Applied Biosystems automated 3730xl DNA analyzer. Among seven H5N6 strains, one named A/Streptopelia decaocto/Jiangxi/G6/2016 (H5N6) was a novel reassortant strain. The coding region of each segment of this strain is base pairs 1 to 1776 (HA), base pairs 1 to 1431 (NA), base pairs 1 to 2341 (PB2), base pairs 1 to 2274 (PB1), base pairs 1 to 2233 (PA), base pairs 1 to 1565 (NP), base pairs 1 to 1027 (M), and base pairs 1 to 875 (NS). The GC contents were calculated using DNAStar v7.1.0 and were 40.82% (HA), 43.16% (NA), 44.86% (PB2), 4.14% (PB1), 43.93% (PA), 47.35% (NP), 47.03% (M), and 44.34% (NS).

The amino acid sequence at the HA cleavage site is RERRRKR/GL, which is characteristic of high pathogenicity in poultry. Additionally, HA had Q222 and G224 (H3 numbering) at the receptor binding site associated with an adaptation to avian-like receptors (9). NA had an 11-amino-acid deletion (positions 58 to 68) in the stalk region, which may be associated with viral adaptation to terrestrial poultry after being introduced from water birds (10). However, there were no mutations for E627K and D701N in PB2 or at positions 26, 27, 30, 31, and 34 in the M2 protein, indicating an inefficiency to replicate in mammals (11) and sensitivity to amantadine (12).

**FIG 1:**
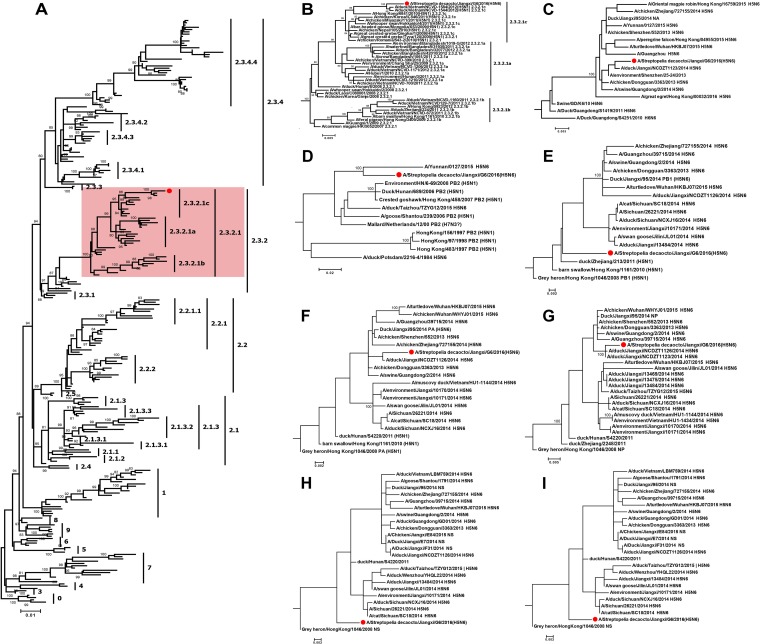
Phylogenetic analysis of Jiangxi clade 2.3.2.1c H5N6 virus. (A) Phylogeny of the HA gene was inferred using the maximum likelihood method with 1,000 bootstrap replicates. Sequence alignment and the inference of phylogeny were conducted using MEGA v6.06. Clades were classified in accordance with the published clade designation. The Jiangxi clade 2.3.2.1c H5N6 virus and clade 2.3.2.1 viruses are highlighted by a dot and colored rectangle, respectively. (B) Phylogenetic relationship of the divergent clade 2.3.2.1 viruses. (C to I) Phylogeny of the NA (C), PB2 (D), PB1 (E), PA (F), NP (G), M (H), and NS (I) genes.

A BLAST search in the GenBank database showed that all the eight genes of Jiangxi H5N6 virus had high nucleotide identities with viruses isolated from terrestrial poultry in China 2014 to 2015 (Table 1). These high nucleotide identities of viruses isolated from wild birds and terrestrial poultry are suggestive of frequent interactions and viral circulation between two species. Specifically, the HA gene shared 99.12% nucleotide identity with the HA gene from four H5N1 viruses. NA, PA, and NP were closely related to those of A/chicken/Jiangxi/NCDZT1123/2014 (H5N6), with identities of 99.37%, 99.24%, and 99.30%, respectively. The PB2 and NS genes shared the closest nucleotide similarity (>99.50%) with the PB2 and NS genes of A/chicken/Jiangsu/2477/2014 (H5N1). For the PB1 and M genes, the highest nucleotide identities were with those of A/pigeon/Zhejiang/112090/2014 (99.85%) and A/duck/Guangzhou/021/2014 (99.51%), respectively. Notably, phylogenetic analysis revealed that this Jiangxi H5N6 virus was a novel reassortant strain, in which the HA gene was found to belong to clade 2.3.2.1c (Fig. 1).

**TABLE 1:** Nucleotide sequencing identities between the novel clade 2.3.2.1c H5N6 virus and nearest homologs in the GenBank database

Gene	Virus	Accession no.	Subtype	Identity (%)
HA	A/bar-headed goose/China/F/2015	MK641386	H5N1	99.21
	A/bar-headed goose/China/70/2015	MK641378	H5N1	99.21
	A/bar-headed goose/China/133/2015	MK641362	H5N1	99.21
	A/whooper swan/Shanxi/17L/2015	KP715064	H5N1	99.21
NA	A/chicken/Jiangxi/NCDZT1123/2014	KP090441	H5N6	99.37
PB2	A/chicken/Jiangsu/2477/2014	KP762511	H5N1	99.53
PB1	A/pigeon/Zhejiang/112090/2014	KU042689	H5N1	99.82
PA	A/chicken/Jiangxi/NCDZT1123/2014	KP090438	H5N6	99.24
NP	A/chicken/Jiangxi/NCDZT1123/2014	KP090440	H5N6	99.30
M	A/duck/Guangzhou/021/2014	KX094409	H5N6	99.51
NS	A/chicken/Jiangsu/2477/2014	KP762516	H5N1	99.77

This detection suggests continuous reassortment and generation of emerging variants. Further investigation and active surveillance are required to detect new AIV variants.

### Data availability.

The genome sequence of Jiangxi clade 2.3.2.1c H5N6 virus has been deposited in GenBank with the accession numbers MN165550 to MN165557.
